# Editorial: Radical Embodied Cognitive Science of Human Behavior: Skill Acquisition, Expertise and Talent Development

**DOI:** 10.3389/fpsyg.2020.01376

**Published:** 2020-07-14

**Authors:** Ludovic Seifert, Keith Davids, Denis Hauw, Marek McGann

**Affiliations:** ^1^CETAPS EA3832, Faculty of Sport Sciences, University of Rouen Normandie, Mont-Saint-Aignan, France; ^2^Sport and Human Performance Research Group, Sheffield Hallam University, Sheffield, United Kingdom; ^3^Institut des Sciences du Sport, Université de Lausanne, Lausanne, Switzerland; ^4^Department of Psychology, Mary Immaculate College, Limerick, Ireland

**Keywords:** sense making, experience, enaction, decision-making, affordances

In this Research Topic, we aim to examine how radical embodied cognitive science (RECS), as a theoretical approach emphasizing the relationship between the body and the environment or ecosystem, may inform conceptions and analyses in the field of movement coordination and skill acquisition and provoke thinking about the full range of psychosocial processes and activities, including experience, mindset, or social interactions.

While other article collections have sought to elucidate key developments of a general conceptual nature, regarding relationships between different schools of thought captured underneath the broad umbrella of radical embodied perspectives, our focus has been more specific and concrete. We wish to bring together examples of theoretical and empirical research, which illustrate how radical embodied cognitive science casts new light on questions of traditionally “higher” functions or directly challenges traditional conceptualizations of those functions. The RECS is often introduced and discussed through 4E concepts (embodied, embedded, enacted, and extended), with some differences explored within and between aligned frameworks (e.g., enactivism, ecological psychology, dynamical system theory, ecological dynamics, phenomenology, etc.). In our Research Topic, we did not restrict RECS to any specific framework, method, or nature of data, but we were open to research diversity about skill acquisition, expertise, and talent development.

The Research Topic is composed of 13 articles representing various types of manuscripts that cover reports of original research to reviews, perspectives, conceptual, and theoretical articles from 41 researchers working in 12 different countries. It is obvious that a community of RECS researchers is spreading over the world and embracing a growing number of domains of human activity.

Rather than introducing each article individually, we have decided to write this editorial by focusing the mind of the future reader on several conclusions that could be drawn from all of these contributions. To do that, we identified five key points in the articles, and we highlight the most relevant to each of us in turn:

(a) Because of the multi-scale nature of body-environment interactions, multilevel methodology and analysis seem to be needed for a complete examination of the relationship between the performer and environment. Montull et al.'s report of original work examining the experience of flow as an emergent property of the behavioral coupling between the performer and environment in performance of a slackline task provides a concrete illustration of how this change in perspective can offer new productive insights.

(b) An in-depth examination of the concept of “representative design” is developed by considering the best way to capture the essence of the activity analyzed. This is suggested by Coste et al., explaining how creativity can be explored, and also by the study of Ra̧czaszek-Leonardi et al., which included cultural artifacts involved in the recognition of difficult experiences.

(c) A growing number of works, reflections, or guidelines that focus on education, prevention, learning, or training have emerged connected to the RECS approach. The study of Button et al. on treading water coordination, the pedagogical framework of Otte et al., and the review of van der Kamp et al. provide clear examples of this, illustrating that radical embodied approaches are not an academic matter but bring with them important practical considerations that affect practice designs.

(d) The “phenomenological” route for understanding body-environment interactions provides additional insights to the strict ecological-dynamical behavioral analysis. This is exhibited by Rochat et al.'s investigations of the experience of learning, linked to the behavioral fluency of climbers observed in a novel and unchanged environment, as well as with the study of van Westen et al. on the effect on the clinician's interactive activity with patients with deep brain simulation.

(e) The “crisis” or “limitations” in certain fields of research because of conceptual, methodological, or theoretical issues might be overcome using a radical embodied approach. This is suggested, for example, by Malinin's article about creativity, by Pacheco et al.'s framework outlining a Search Strategies Approach to skill acquisition (SSA), or by Raab and Araújo's exploration of dialogue and the place of representations in RECS.

This special issue is rich, diverse, and provides inspiring methods and models that RECS principles bring forth. We are sure that attentive readers will find new opportunities to enrich their own research and professional activity and may uncover a new world of acting in research and practice.

## Author Contributions

All authors listed have made a substantial, direct and intellectual contribution to the work, and approved it for publication.

## Conflict of Interest

The authors declare that the research was conducted in the absence of any commercial or financial relationships that could be construed as a potential conflict of interest.

